# The association between anxiety and internet addiction among left-behind secondary school students: the moderating effect of social support and family types

**DOI:** 10.1186/s12888-024-05855-4

**Published:** 2024-05-30

**Authors:** Siwei Miao, Lu Xu, Sihong Gao, Cuiping Bai, Yan Huang, Bin Peng

**Affiliations:** 1 Centre for Medical Big Data and Artificial Intelligence, Southwest Hospital of Third Military Medical University, Chongqing, 400038 China; 2 School of Pharmacy, Chongqing Medical and Pharmaceutical College, Chongqing, 401331 China; 3 Chongqing Fuling District Center for Disease Control and Prevention, Chongqing, 408000 China; 4 Xiushan Traditional Chinese Medicine Hospital, Chongqing, 409900 China; 5 Chongqing Traditional Chinese Medicine Hospital, Chongqing, 400021 China; 6 Department of Health Statistics, School of Public Health, Chongqing Medical University, Chongqing, 400016 China

**Keywords:** Anxiety, Internet addiction, Social support, Family types, Left-behind secondary students

## Abstract

**Background:**

The left-behind children (LBC), children and adolescents aged 0–18 whose parents have migrated for economic purposes for extended periods exceeding three months, present a unique social concern. These children remain in their place of household registration, often under the guardianship of relatives, while receiving compulsory education. LBC with growing Internet addiction (IA) have made it urgent to take a close look at the mechanisms and effective interventions for them. Anxiety has been proven to be correlated with IA in adolescents; however, the mechanisms of addiction in this population are less well-fully grasped. Based on the current theories and empirical results, the study examined whether and how social support (SS) and family types moderated the associations between anxiety and IA among left-behind secondary students.

**Methods:**

Stratified cluster sampling survey. A questionnaire was administered to 5290 secondary school children (2553 classified as left-behind) to explore the relationships between anxiety, IA, left-behind types, family types, and social support. This cross-sectional study employed a stratified cluster sampling survey of students in the ethnic areas of southeast Chongqing. The study sought to appraise the relationships between anxiety and IA in different types of left-behind children and to assess the potential moderating effect of SS on the relationship among the population and its family types differences.

**Results:**

The relationship between anxiety and IA was moderated significantly by social support and family types. Notably, the impact of social support on the moderating effect between IA and anxiety varied among students from both family types. For students from families where both parents had migrated, social support weakened the association between IA and anxiety. Conversely, for students from single-parent families where the parent had migrated, social support seemed to strengthen the relationships between these two issues.

**Conclusions:**

The moderating effects of SS on the relationship between anxiety and IA differs based on family type among various groups of left-behind secondary students. Gaining insights into the IA mechanisms can guide the development of targeted intervention strategies aimed at minimizing IA among diverse groups of left-behind students.

## Introduction

Amidst the transformation of Chinese society, left-behind children (LBC) epitomize a unique cohort emerging parallel to the wave of urbanization, directly linked to the migration of rural laborers into urban centers [1]. LBCs frequently experience less communication with their parents, a circumstance that undermines a fundamental pillar of psychological development of children and adolescents. The parent–child dynamic and the quality of family education hold significant sway over adolescents’ growth trajectories [2]. Prolonged parental absence can weaken parent–child bonds and disrupt normative family types and functions. These disruptions can leave LBCs more prone to negative affective states and a range of negative psychological and behavioral issues [3, 4]. The research found that LBC had a higher detection rate of Internet addiction (IA) [5], increased risk of anxiety [6], and more inadequate access to social support [7]. Left-behind secondary school students find themselves in a particularly sensitive developmental phase marked by rapid physiological and psychological transformation. This renders them more susceptible to external effects with the potential to exacerbate psychological vulnerabilities [8]. Notably, the absence of parental care may drive students to seek solace or distraction in the online world, thereby increasing their risk for internet addiction [9–11]. By the end of 2020, above 1.4 million secondary school students were left behind in rural [12]. The mental health condition associated with Internet addiction in this population necessitates urgent research and intervention.

At present, there is no single agreed-upon definition for IA [13]. The study supports that IA is a distinct pathological behavioral and cognitive maladaptive psycho-behavioral disorder resulting from excessive Internet use due to uncontrolled behavioral impulses to be online without the effect of addictive substances [14]. Chinese researchers Gao and Chen demonstrate that Internet addiction is a dependent behavior caused by psychological mechanisms [15]. The studies found that IA causes a certain degree of negative impact on secondary school student’s academic development and mental health and is an influential driver of boredom and withdrawal from school for students currently [16, 17], which leads to the rising occurrence of depression and loneliness [18] even to the emergence of non-suicidal self-injurious behavior [19]. A statistical analysis from December 2021 highlighted that 13.3% of internet users between the ages of 10 to 19 are affected [20]. Given the apparent trend of a younger age for web use, the harm of Internet addiction, and the still challenging problem of left-behind middle school students, it is essential to explore the specific mechanism of the role of IA among population.

### Anxiety and internet addiction

Based on the compensatory internet use theory (CIUT), individuals experiencing high levels of social anxiety might seek compensatory satisfaction through the internet with the intention of mitigating negative emotions, thereby potentially leading to the IA [15, 21, 22]. Anxiety is a common negative emotional issue in the adolescent population, and the detection rate is on the rise annually [23]. During this period, the teenager is prone to anxiety condition due to the low-income family environment and weak levels of family cohesion [24, 25]. Moreover, the developmental course of emotional problems is protracted, potentially giving rise to other symptoms [26]. The relationship between anxiety and IA has been extensively explored in the context of general adolescent populations [27, 28]. Empirical evidence robustly demonstrates a significant correlation between anxiety and internet addiction [29]. However, the current relationship between the left-behind secondary school students accompanying anxiety disorders and IA is unclear. In accordance with the CIUT, individuals may resort to problematic internet overuse to alleviate or circumvent feelings of anxiety in their offline lives, or as a reaction to unmet psychological needs, thereby contributing to IA.

### Social support, anxiety and internet addiction

Social support (SS) is vital for maintaining their mental health [30]. According to the paradigm of deteriorating SS, surroundings, and stressors such as family dysfunction and peer rejection might result in a decline in social support expectancies and interpersonal conflict. Due to impaired parental functioning, the left-behind population with inadequate social support may develop an IA [31]. Davis proposed a cognitive-behavioral model for IA, dividing influencing factors into distal necessary conditions and proximal sufficient conditions. The latter primarily includes maladaptive cognitions and lack of SS [32]. Studies have established a correlation between SS and IA [33], indicating that adolescents with limited social support struggle with social adjustment, lack the necessary support from their surroundings [34], and are more inclined to seek confirmation and support through the internet [35]. Conversely, those surrounded by a strong support network are less likely to engage in behaviors associated with IA [36]. Fan verified that social support significantly impacts Internet addiction in Chinese left-behind students [4]. Children experiencing anxiety tend to be more reserved in social situations due to a fear of rejection or criticism [37]. This hesitancy can impede their ability to seek out support during challenging times, leading to a lower capacity to secure real-life SS [38].

Person-context interactions theory relate psychological phenomena to the dynamic relationship between the individual and the environment [39]. Anxiety and SS represent intrinsic susceptibility and the external environments of individuals, respectively. The development of IA among left-behind students may be the joint result of the two factors. Empirical studies indicate that left-behind students often grow up deprived of sufficient social resources [40]; facing personal hurdles or setbacks, they turn to the internet as a means to mitigate their anxiety and search for a sense of belonging that is missing from their real-life interactions.

### Left-behind types differences in social support, family types, anxiety, and internet addiction

Ecological Systems Theory (EST) presents an indispensable framework for analyzing child development. Its central premise is that an individual’s environment (e.g., family, school, community, etc.) functions as a dynamic social ecosystem, characterized by reciprocal interaction and evolution between the individual and their surroundings [41]. The multifaceted developmental challenges encountered by children left behind in rural areas are due to a complex combination of factors – individual, familial, related to parental work environments, and embedded in broader sociocultural forces. EST sheds light on the effects at different hierarchical levels: microsystem (the surrounding environment with which individuals interact closely), mesosystem (the interaction of elements in the bit system), outer system (the external environment), and macrosystem (the broader social system). This model offers a novel theoretical foundation for our study, which evaluates the effect of family type and social support on the association between anxiety and IA. Family constitutes the microsystem where adolescents’ psychological development is shaped. The family relationships and parenting practices exert a significant effect on adolescents’ self-esteem and mental well-being. The left-behind population, separated from one or both parents for a long time, is more prone to psychological problems in growth and cannot fit well into the interaction environment [42]. This problem also includes the development of IA, and related studies have found that not living with parents is significantly associated with the detection of IA [43]. The family types have been categorized into intact families (families with two parents and children) and non-intact families (divorced parents, one of whom is deceased). Based on the mesosystemic level, individuals with incomplete family types are more prone to close themselves off to the online world due to the family’s malfunctioning and the family structure’s deficiency, which is not conducive to home-school cooperation and affects the socialization development of the left-behind group. Gan et al. found family risk factors linked to IA [44]. The outer system emphasizes the significant role of social support, which indirectly modulates left-behind secondary school students’ acclimation to society. Robust social support networks can serve as a protective factor against IA. In light of these considerations, it seems plausible that the family types and social support experienced by left-behind secondary school students exhibit a complex and potentially significant relationship with the rate of IA.

Anxiety, SS, family types, and IA were found to demonstrate significant discrepancies among different classifications of left-behind children. This study distinguishes between intact families (families with two parents and children) and non-intact families (families where parents are divorced or one parent is deceased). In contrast to those from intact families, individuals from non-intact families with anxiety symptoms may exhibit a greater likelihood to seek psychological solace through inordinate internet usage, potentially terminating IA [45]. Familial disruptions characteristic of non-intact families frequently precipitate reduced self-esteem, interpersonal challenges, and a paucity of social support in affected students when compared to those from intact families [46]. However, to our knowledge, the moderating function of social support in the relationship of family type differences, IA, and anxiety remains to be rigorously substantiated. Wang and Lu et al. identified a significant correlation between IA and the specific parent who has migrated [47]. Moreover, studies have indicated differences in the expression of anxiety across different left-behind family types [6, 48]. While extant research and theory propose that a supportive family environment and robust social networks can mitigate the effect of anxiety on IA, additional research is necessary to corroborate this hypothesis.

**Fig. 1:**
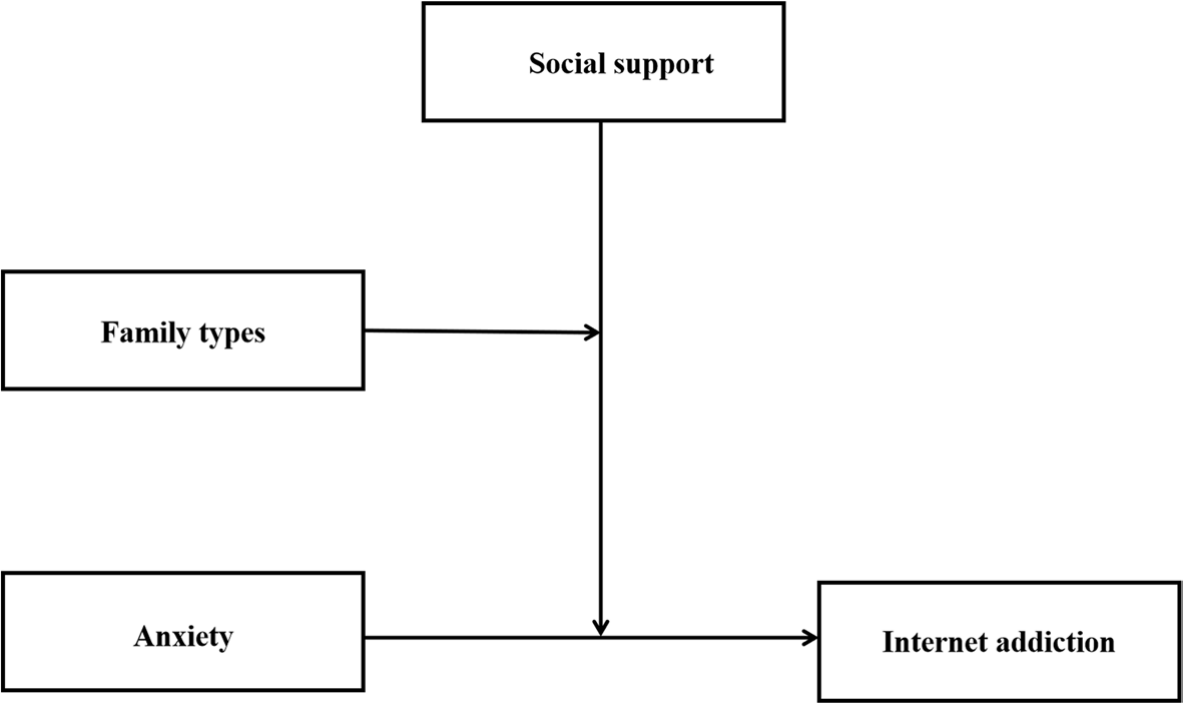
Diagram of the theoretical dual-moderated model

Additionally, Tao et al. discovered a moderating effect of the parent-migrant type on the link between personality traits and the psychological well-being of the population [49]. Xu et al. have found that students with single-parent families faced low perceived social support levels [50]. Hence, according to the current theories and empirical results, the hypotheses of a dual-moderation model is proposed: (1) anxiety is positively associated with IA; (2) SS moderates the relationship between anxiety and IA; (3) SS and family type simultaneously moderated the relationship between anxiety and IA. A theoretical hypothesis model is shown in Fig. 1.

## Materials and methods

### Subjects and procedure

The cross-sectional study was conducted in ethnic areas of southeast Chongqing, Xiushan Autonomous County and Shizhu Autonomous County in May 2021. Our Hospital Human Research Ethics Committee approved ethics permission to implement this project (Approval number: 2019-ky-ks-HY-1). And the research received support from participating schools, which facilitated the centralized distribution of the questionnaire. Students aged 12 to 18 from township schools in the ethnic regions of southeast Chongqing (Xiushan Autonomous County and Shizhu Autonomous County), were selected through a stratified cluster random sampling method for this study. In each district and county, 5 to 10 township schools were chosen at random to serve as the sample schools, and all students in these schools were invited to participate in the on-site survey. Consent was obtained from both students and their guardians for voluntary participation, with the option for non-participation available at any time. Prior to the survey, researchers thoroughly explained the questionnaire instructions and were available for further clarification during its completion. The questionnaire was completed in the classroom throughout the school day. The questionnaire and the scales was administered in a classroom setting during the school day, taking between twenty to thirty minutes to complete. The survey contained questions about Internet addiction, anxiety, and social support. The criteria for inclusion in the study were as follows: being between the ages of 12 and 18, enrolled as a secondary school student, and indicating a willingness to engage with the survey and complete it diligently.Subjects who did not complete the survey or failed to meet consistency checks were excluded from the study. The intention behind the lie detection measure was to identify and exclude participants who were not providing sincere responses. Furthermore, the investigation with obvious logical errors was eliminated. This included individuals who reported that one or both parents had been employed outside of their hometown, yet subsequently chose “No period of working away” when asked about the length of time their parents spent working away from home.

**Fig. 2:**
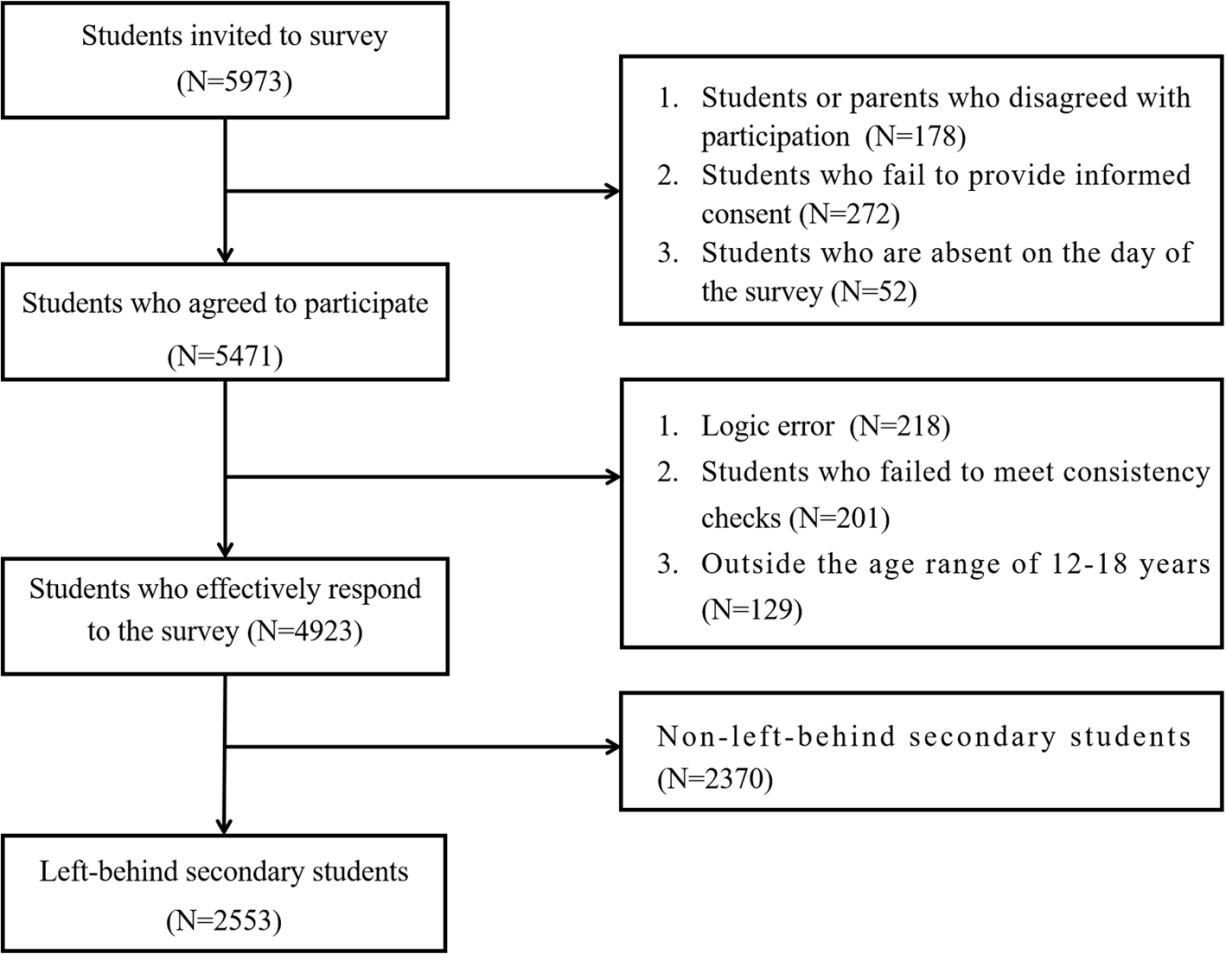
The flow chart of enrollment of subjects in the study

Preliminary findings from the survey indicated that the incidence of IA among Chongqing’s adolescents stands at approximately 25%. Therefore, the study included a total of 5973 secondary school students. Of these, 4923 submitted valid questionnaires (82.42%). The participants’ ages ranged from 12 to 18 (${\text{M}}_{\text{age}}=14.63$,$\text{SD}=1.39$), with a gender distribution of 2483 males (50.44%) and 2440 females (49.56%). The study identified 2553 participants (48.25%) as left-behind secondary students, with a gender split of 50.18% (1281) males and 49.82% (1271) females among them. In addition, it was found that 1156 cases (45.28%) with single-parent migration and 1397 cases (54.72%) form both-parent migrant families. The flowchart of enrollment of subjects is showed in the Fig. 2.

### Sample size

The determination of the total sample size necessary for this survey was guided by a formula recommended by the World Health Organization [51].

$$\text{n}=\frac{{Z}^{2}P(1-P)}{{d}^{2}}\times deff$$

*Z* was 1.96.* P* was estimated based on the prevalence of IA among youth in Chongqing was about 25% according to the results of a previous study. *d*, the allowable absolute error level, was 2%. Design effect (*deff*) was 2. The minimum sample size calculated for this study was approximately 4322 school students aged 12–18 years in rural schools. A total of 5973 secondary school students were surveyed in this study, and 4923 valid questionnaires were returned, with an effective rate of 82.42%.

### Measures

#### Sociodemographic information form

The demographics of the left-behind students in the secondary school were collected by a self-designed general information questionnaire, including gender, grade, and family situation, which included the left-behind type, family type, family location, and family economic status.

#### The internet addiction

Young developed the Internet Addiction Test (IAT) based on the pathological gambling diagnostic criteria included in DSM-IV to class Internet addiction of left-behind students [14]. And the Chinese version of the scale was used to determine the population’s degree of IA [52]. The IAT consists of 20 items, each assessed on a 1 to 5 scale, with scores ranging from 20 to 100. The point represents the severity of IA. IAT is one of the most extensively used scales for assessing IA [53, 54]. It has demonstrated strong reliability and validity in studies involving Chinese adolescents [55]. Furthermore, the IAT evaluates a broad concept of IA rather than focusing on particular manifestations like Internet gaming disorder and problematic smartphone use. This approach enables a more holistic evaluation of IA [56]. The results (Cronbach’s α = 0.91) showed great reliability in the study.

#### Social support

The Adolescent Social Support Rating Scale (ASSRS) by Ye & Dai [57] was used to evaluate the SS of stay-at-home secondary school adolescents. Reckoning Xiao’s theoretical model of social support [58] and Ye and Dai’s [57] design, the scale consisted of 17 questions measuring three dimensions. On a 5-point Likert scale, the scale spans from 1 to 5, with higher marks suggesting positive ranks of social support. The dependability of the scale is excellent since its Cronbach’s α coefficient of the scale is 0.96.

#### Anxiety

The 7-item Generalized Anxiety Disorder scale (GAD-7) was assessed among left-behind students. The scale was created as a self-reporting scale for identifying anxiety and grading the intensity of anxiety symptoms [59]. Higher scores on the 21-overall-point scale indicated increased levels of anxiety. Each of the seven items on the scale ranged from 0 (suggesting “not at all”) to 3 (showcasing “almost every day”). The reliability of the scale was fair, as evidenced by a Cronbach’s α value of 0.92.

#### Data analysis

The Statistical Package for the Social Sciences (SPSS) version 26.0 was used to clean and analyze the data. First, a two-way analysis of variance was performed to test the group difference in the three essential variables, which were IA, social support, and anxiety, between gender, left-behind type, and family types; analysis data was displayed by means and standard deviations. Second, the level of correlation of the selected continuous random variables was investigated by picking the Pearson correlation coefficient to investigate. Moreover, the study used model 3 in PROCESS macro version 4.0, developed by Hayes [60], to analyze moderation using a hierarchical regression model, with gender and online time as control variables, anxiety as independent variables, social support and family types as moderating variables, and Internet addiction scores as dependent variables for the two types of stay-at-home types, respectively. Last, a simple slope analysis of the moderating effect of social support as are family types was concluded in the study. The mediation effect was tested using the bootstrap method with a sample size of 5000. A significant moderating effect is presented if the 95% confidence interval (CI) does not involve zero.

## Results

### Demographic characteristics

For the population of left-behind secondary school students, there is a statistically significant difference in anxiety scores between males and females, with females displaying notably higher scores than males (*p* < 0.01). Interestingly, students from incomplete families reported higher anxiety levels compared to their peers from intact families (*p* < 0.001). There were significant differences in social support by gender, age, and left-behind types. Female students displayed significantly higher scores in social support than males (*p* < 0.05). Older left-behind secondary school students had better social support scores (*p* < 0.01). Left-behind middle school students who primarily use the internet for entertainment make up 78.97% of the population. Notably, these students display significantly higher internet addiction scores compared to those who primarily use the internet for academic research purposes (*p* < 0.001). The group with non-intact families received better SS than intact families (*p* < 0.001). There were statistically significant differences in IA in terms of age and family type. IA scores were higher in the older age group than in the younger age group (*p* < 0.05). Notably, IA scores were higher in left-behind secondary school students in non-intact families than in the intact family group (*p* < 0.05). Detailed results could be found in Table 1.

**Table 1:** The demographic characteristics among left-behind secondary students (*N* = 2553)

Variable	Number	Proportion	t-value	Anxiety	SS	IAD
	(Total, *n* = 2553)	(%)	*P* value			
Gender						
Male	1272	49.82		4.50 ± 4.80	65.65 ± 15.68	42.16 ± 13.85
Female	1281	50.18		5.07 ± 5.03	63.94 ± 15.83	41.49 ± 13.43
			*t* value	2.935	-2.264	-1.248
			*P value*	0.003	0.024	0.212
Age(years)						
12–15	1800	70.51		4.78 ± 4.96	65.26 ± 15.97	41.38 ± 13.91
15–18	753	29.49		4.80 ± 4.83	63.16 ± 15.18	42.87 ± 12.93
			*t* value	-0.80	3.071	-2.509
				0.936	0.002	0.010
Activity on Internet						
Study	537	21.03		4.16 ± 4.772	68.16 ± 15.90	36.05 ± 11.69
Entertainment	2016	78.97		4.95 ± 4.95	63.70 ± 15.60	43.36 ± 13.72
			*t* value	-3.308	5.856	-12.39
			*P value*	< 0.001	< 0.001	< 0.001
Average Time Spent Online Use Per Day						
< 6	1999	78.30		4.27 ± 4.63	65.85 ± 15.48	38.73 ± 12.22
≥ 6	554	21.70		6.62 ± 5.48	60.25 ± 16.01	52.99 ± 12.59
			*t* value	-29.22	7.48	-24.13
			*P value*	< 0.001	< 0.001	< 0.001
Intact family	2230	91.27		4.62 ± 4.81	65.18 ± 15.56	41.62 ± 13.39
Incomplete family	323	8.73		5.94 ± 5.50	60.91 ± 16.69	43.22 ± 15.24
			*t* value	4.541	-4.565	1.976
			*P value*	< 0.001	< 0.001	0.048
Left-behind type						
Students with one-parent migration	1156	45.28		4.86 ± 4.97	64.99 ± 15.88	41.75 ± 13.45
Students with two-parent migration	1397	54.72		4.72 ± 4.88	64.35 ± 15.67	41.88 ± 13.80
			*t* value	0.754	1.019	-0.237
			*P value*	0.451	0.308	0.812

*SS* Social support, *IAD* Internet addiction

We used analysis of ANOVA to examine how SS, anxiety, and IA levels varied by leftover group, gender, family types, and the interaction between parent migration type and gender and family type (Table 2).

**Table 2:** The results of Multivariate ANOVA

Variable	Anxiety		SS		IAD	
	F-value	*P* value	F-value	*P* value	F-value	*P* value
Gender	8.617	0.003	5.123	0.024	1.558	0.212
Family type	20.617	< 0.001	20.837	< 0.001	3.903	0.048
Left-behind type	0.569	0.4551	1.038	0.908	0.056	0.812
Gender*Left-behind type	6.456	0.011	0.514	0.473	0.757	0.385
Family type*Left-behind type	0.160	0.689	0.340	0.560	0.317	0.573

*SS* Social support, *IAD* Internet addiction

The main effect of gender on anxiety was significant (F_1,2549_=8.617, *p*<0.001), with female students indicating greater anxiety levels than male students. Regarding anxiety scores, the effect of the left-behind category did not exhibit statistical significance (*p* > 0.05). However, the interaction between gender and the left-behind category was significant (F_1,2549_ = 6.46, *p*<0.05). In cases where both parents had migrated, the analysis indicated significant gender differences, with females experiencing more severe anxiety levels than males (*p* < 0.001). In addition, the effect of family types on anxiety levels was significant (F_1,2549_ = 20.617, *p*<0.001), with students from incomplete families reporting significantly higher anxiety levels than those from intact families.

The results revealed that the main effect of gender on social support was significant (F_1,2549_ = 5.123, *p*<0.05), and the score of social support was significantly higher for males than for females; the main effect of family types on social support was significant (F_1,2549_ = 20.837, *p*<0.001), and the social support score was greater for intact families than for incomplete families. The parent migration groups’ primary effect was insignificant (*p* > 0.05).

Regarding IA, the main effect of family types was significant (*p* < 0.05), whereas the main effects of gender and parent migration status were found to be insignificant (*p* > 0.05).

### Correlation analysis

The analysis exploring the connections among overall scores for IA, anxiety, and social support, including its various facets, highlighted significant relationships across all principal factors, as illustrated in Table 3. A significant positive association was identified between anxiety and IA in secondary school students who were left behind. Moreover, there was a strong negative correlation between IA and social support, including its three dimensions, in students residing with their families.

**Table 3:** Descriptive statistics and correlations among the essential variables

Variables	*M*	$SD$	1	2	3	4	5	6
1 Internet Addiction	41.82	13.64	1					
2 Anxiety	4.78	4.92	0.45^**^	1				
3 Social Support	64.64	15.77	-0.30^**^	-0.38^**^	1			
4 Objective support	23.82	5.67	-0.26^**^	-0.35^**^	0.90^***^	1		
5 Subjective support	18.79	5.09	-0.30^**^	-0.37^**^	0.92^***^	0.77^***^	1	
6 Utility of support	22.03	6.33	-0.28^**^	-0.35^**^	0.95^***^	0.78^***^	0.82^***^	1

*p* < 0.05

^**^p < 0.01

^***^p < 0.001

### Dual-moderation model analysis

Although the results of the above descriptive statistical analysis found no significant differences between the two groups of the types of left-behind in IA, considering that previous studies confirmed the differences in the detection of IA by left-behind [47, 61, 62], the present study still divided into single-parent left-behind and two-parent left-behind to test the model effects. To examine the effect of anxiety on IA and the moderating effects of social support and family types, gender, activity on internet, and network access time were treated as dummy variables as control variables, anxiety as an independent variable, social support, and family types as moderating variables, and Internet addiction as a dependent variable to performed hierarchical regression models for two strata of the left-behind categories of secondary school students, respectively. Before entering the model, the continuous anxiety and social support variables were centralized. The variables were entered into the model in the following steps: First, gender and age were included in the model to control; second, the main effects of the variables were examined; third, the second-order interaction terms of the model were assessed (Anxiety × Social support, Anxiety × Family types, Social support × Family types; and fourth, the third-order interaction terms were tested (Anxiety × Social support × Family types). To investigate the trend of the moderating effect, a simple slope test was used to cope with the significant interaction term in the regression model. And social support was divided into high- and low-level groups based on the mean plus- minus one standard deviation to plot the interaction effect on the efficacy to reveal the dual moderating effect of social support and family types.

### Association between anxiety and *IA* among left-behind secondary students with single-parent migration: the moderating effect of social support and family types

**Fig. 3:**
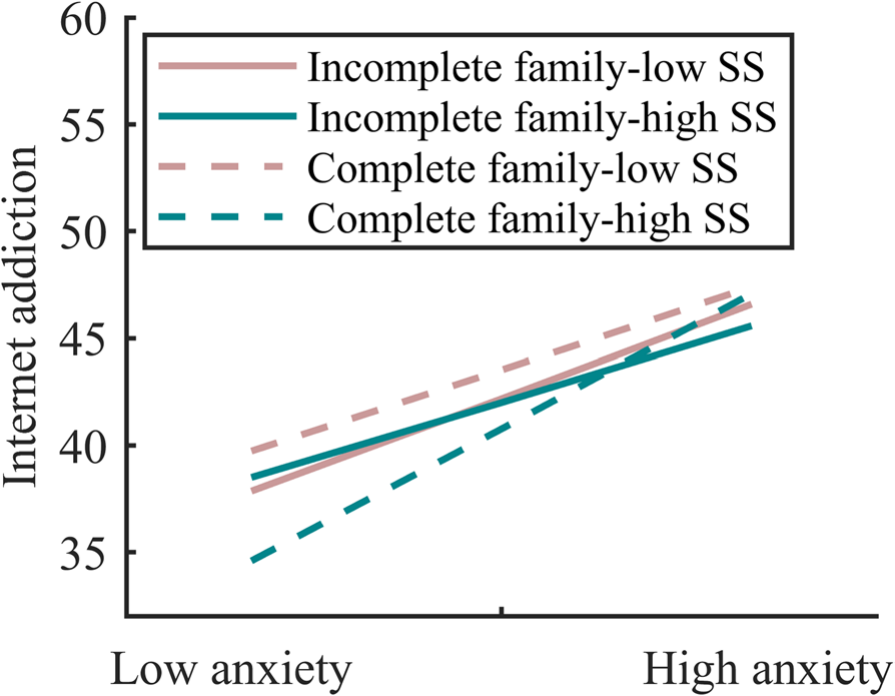
Interaction of anxiety, social support and family types on Internet addiction in the left-behind students with one-parent migration. Note: SS means social support

Table 4 displays the effects of each variable and interaction terms on Internet addiction among single-parent migrating students. Model 4 demonstrated a positive effect of anxiety on Internet addiction (*t* = 4.79, *p* < 0.001), a non-significantly passive effect of social support (*t* = -0.01, *p* > 0.05), and family types (*t* = 0.06, *p* > 0.05). The third-order interaction had statistical significance (*t* = 2.36, *P* < 0.01), as it was not at a zero-point estimate interval within the bootstrap 95%CI (0.01–0.05). Further simple slope analysis was conducted, revealing that for left-behind middle school students from intact families, regardless of whether they were in the low or high SS group, there was a significant positive correlation between anxiety and IA (*simple slope* = 0.77, *t* = 8.75, *p* < 0.001); (*simple slope* = 1.28, *t* = 11.34, *p* < 0.001). According to Dawson and Richter [63], among left-behind middle school students from intact families, examining the correlation effects of anxiety on IA across varying levels of SS. The results demonstrated that the related coefficient of anxiety on IA significantly differed depending on the extent of social support (*t* = 2.08, *p* < 0.05). As illustrated in Fig. 3, the Internet addiction scores of intact family left-behind middle school students at low social support conditions were all higher than the high level of SS, but the protective effect of high SS on their Internet addiction was diminished when they were in a high anxiety situation. In environments characterized by both low and high SS, a significant positive correlation is observed between anxiety levels and internet addiction among middle school students from non-intact families who are left behind (*simple slope* = 0.88, *t* = 5.34, *p* < 0.001; simple slope = 0.72, *t* = 2.99, *p* < 0.01). However, the efficacy of this correlation did not show any significant differences when comparing the low social support group with the high social support group (*t* = -0.11, *p* > 0.05).

**Table 4:** The moderating effect test of SS and family types among leftover secondary students with one-parent migration

Variable	Internet Addiction							
	Model 1		Model 2		Model 3		Model 4	
	$\beta$	*t*	$\beta$	*t*	$\beta$	*t*	$\beta$	*t*
Gender	0.06	0.08	0.21^**^	0.32	-0.10	0.16	0.09	0.14
Network use purpose	6.02^***^	6.85	5.64^***^	7.05	5.55^***^	6.96	5.54^***^	6.95
Network-access time	13.52^***^	15.81	10.69^***^	13.39	10.70^***^	13.45	10.66^***^	13.43
Anxiety			0.93^***^	13.39	0.93^***^	5.85	0.80^***^	4.79
SS			-0.07^***^	-3.33	-0.03	-0.55	-0.01	-0.11
Family types			-0.19	-0.22	-0.36	-040	0.06	0.06
Anxiety * SS					0.01^**^	3.09	-0.01	-0.68
Anxiety * Family types					0.07	0.41	0.22	1.20
SS * Family types					-0.06	-1.01	-0.08	-1.41
Anxiety * SS*Family types							0.02^**^	2.36
∆*R*^2^	0.223		0.137		0.007		0.003	
*F*	110.127^***^		82.044^***^		4.054^**^		5.559^*^	

*SS* Social support

^*^P < 0.05

^**^P < 0.01

^***^P < 0.001

### Association between anxiety and *IA* among left-behind secondary students with both-parent migration: the moderating effect of social support and family types

**Fig. 4:**
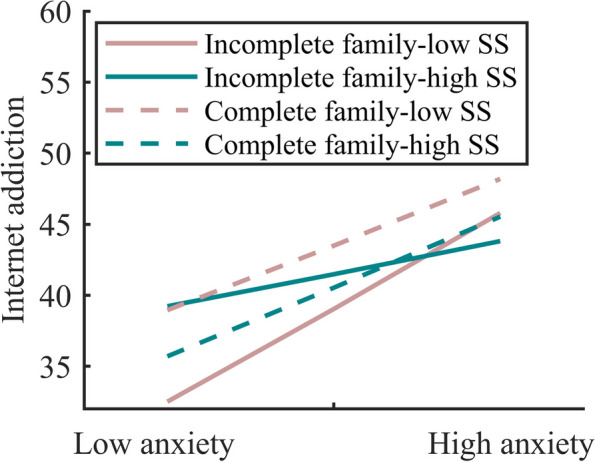
Interaction of anxiety, social support and family types on Internet addiction in the left-behind students with both-parent migration. Note: SS means social support

The results of the effects of each variable and its interaction term on IA among bi-parental left-behind secondary school students are reported in Table 5. The results revealed a significant tripartite interaction between anxiety, SS, and family type, with a bootstrap 95% CI excluding 0 (0.01–0.05). This validated the high-order moderation model, suggesting a family type-dependent difference in the moderating role of SS on anxiety and IA in both-parent left-behind secondary students.To further investigate the moderating differences, a simple slope test of the association of anxiety with IA was performed among students by family type, based on the high and low-group conditional values of social support (Fig. 4). For intact families, anxiety was a significant − positive associated factor of IA among left-behind secondary school students with low and high levels of SS (*simple slope* = 0.96, *t* = 12.19, *p* < 0.001); (*simple slope* = 1.02, *t* = 9.42, *p* < 0.001), however, the difference between the two effects was not significant (*t* = 1.53, *p* > 0.05). For incomplete family types, anxiety significantly and positively related to IA among the students at low levels of SS (*simple slope* = 1.38, *t* = 6.37, *p* < 0.001), whereas at high levels, there was no association between anxiety and IA (*simple slope* = 0.48, *t* = 1.40, *p* > 0.05). Considering low levels of SS, the effect of anxiety on IA among intact families was weaker (simple slope = 0.96, *t* = 12.19, *p* < 0.001) than non-intact families (*simple slope* = 1.38, *t* = 6.37, *p* < 0.001) (*t* = -3.34, *p* < 0.05).

**Table 5:** The moderating effect test of SS and family types among leftover secondary students with two-parent migration

Variable	Internet Addiction							
	Model 1		Model 2		Model 3		Model 4	
	*β*	*t*	*β*	*t*	*β*	*t*	*β*	*t*
Gender	1.36^*^	2.07	2.50^***^	4.24	2.46^***^	4.17	2.39^***^	4.06
Network use purpose	5.56^***^	6.94	4.42^***^	6.14	4.56	6.30	4.62^***^	6.40
Network-access time	13.70^***^	17.12	11.21^***^	15.44	11.20^***^	15.39	11.18^***^	15.42
Anxiety			1.01^***^	14.85	1.32^***^	5.91	0.93^***^	3.69
SS			-0.09^***^	-4.11	-0.01	0.01	0.08	1.02
Family types			0.56	0.58	0.66	0.65	1.75	1.66
Anxiety * SS					-0.01	0.73	-0.03^**^	-3.33
Anxiety * Family types					-0.36	-1.55	0.06	0.24
SS * Family types					-0.09	-1.24	-0.17^*^	-2.18
Anxiety * SS *Family types							0.03^**^	3.30
∆*R*^2^	0.211		0.163		0.001		0.005	
*F*	123.931^***^		120.105^***^		1.078		10.881^**^	

*SS* Social support

^*^P < 0.05

^**^P < 0.01

^***^P < 0.001

## Discussion

Drawing upon comparisons between left-behind secondary school students experiencing single-parent and two-parent migration, this study employed an EST model to describe the characteristics of IA in this population. It evaluated the correlation of anxiety and social support with respect to students’ IA. Additionally, the study analyzed the moderating discussed of SS and family types upon the relationship between anxiety and IA. Findings demonstrated that anxiety served as a robust correlated factor of IA across all categories of currently left-behind secondary school students (single and two-parent). Moreover, SS and family type displayed a co-moderation effect on the anxiety-IA relationship, with their effect contingent upon the particular left-behind status. In addition, the study discovered a 23% IA prevalence among surveyed rural left-behind secondary school students – exceeding rates in other regions [64, 65]. This discrepancy may be ascribed to variables such as living environment, familial socioeconomic circumstances, and divergent patterns of internet utilization [66, 67].

Analysis of correlations indicated a negative association between social support and IA. This suggests that left-behind secondary school students with deficient SS could exhibit an increased detection rate of IA. This finding resonates with Mo et al.’s research, which posits that robust social support networks may attenuate internet-dependent behaviors [68]. This result could arise from the capacity of strong social relationships to mitigate the negative effects of stressful events [69, 70]. Students with significant social support systems may possess superior coping mechanisms for anxiety, derive greater fulfillment from real-world interactions (both material and psychological), and have a greater capacity to disengage from potentially addictive online environments. Surprisingly, there were no significant differences in social support observed among different categories of left-behind students. However, students experiencing single-parent migration tended to benefit from greater social support compared to those with both parents absent due to work. This observation is consistent with previous research [71]. One potential explanation is that a parent remaining at home in single-parent migration scenarios can offer immediate care and emotional stability. Conversely, secondary school students with both parents absent may confront unique and complex challenges. In addition, positive family function (characterized by strong parent–child bonds, parental engagement, etc.) may cultivate greater SS from sources such as teachers, peers, and extended family, potentially reducing the likelihood of psychological distress. It appears that single-parent left-behind populations may experience more healthy family function.

This study indicated a negative correlation between SS and anxiety, implying that left-behind secondary students who experience higher levels of anxiety tend to perceive a lower degree of SS. These findings are consistent with the research conducted by Yang and Lu [38]. Their study, including 710 junior high school students nationally, similarly demonstrated a negative correlation between SS and social anxiety in the demographic of left-behind children. The relationship between SS and anxiety is further corroborated by both longitudinal and cross-sectional studies. Research indicates that inadequate SS accessibility can be a contributing factor to an increased susceptibility to anxiety. Scardera et al., in their longitudinal survey of 1,174 Quebec teenagers, discovered that higher perceived SS corresponded with reduced anxiety symptomatology [72]. Reciprocally, Scanlon et al. evidenced that social anxiety resulted in decreased peer SS among an adolescent population [73].

In accordance with the research’s first hypothesis and consonant with previous findings, this study demonstrated that anxiety exhibited a positive associated relationship with IA among left-behind secondary school students. This highlights anxiety as a significant positive related factor for this population (H1) – higher anxiety scores were associated with intensified IA ratings [74, 75]. Mirroring past studies of different family types [76, 77], incomplete families displayed increased anxiety scores compared to those in joint-custody settings. No statistically significant differences were observed in how anxiety affects IA across the various categories of left-behind secondary school students. However, the study indicated that anxiety in the two-parent migration group exerted a more significant effect on IA problems than anxiety in the single-parent migration group. Anxious individuals deficient in robust family support systems may seek a sense of belonging in the online world to alleviate academic and interpersonal stressors [78, 79]. This could explain the proclivity of these students to utilize the internet as a mechanism for managing anxiety and loneliness. Additionally, left-behind adolescents whose parents migrate for work exhibit increased susceptibility to psychological distress [80, 81]. At the same time, weakened parental supervision increases the potential for the development of problematic behaviors [82], particularly for left-behind secondary school students with both parents absent. The lack of parental involvement and potentially dysfunctional families (such as strained parental relationships or divorce) impede these students’ ability to secure parental support and affection when confronting anxiety – potentially exacerbating behavioral problems [83, 84].

In this research, the presence of social support was found to moderate the correlation between anxiety and IA (H2), with this effect varying by family type (H3). Study results indicated that among left-behind students with two-parent migration, anxiety under conditions of low social support predicted IA in the non-intact family group. In contrast, where high social support levels existed, anxiety did not reliably associate with IA. This implies family factors and a robust social support environment may attenuate the relationship between anxiety and IA. For students experiencing single-parent migration in intact families, however, social support’s buffering effect was not apparent. The researchers surmise that IA development in students from intact families is primarily attributable to traits such as anxiety. Conversely, external factors such as social support and family characteristics may lessen effect of anxiety on IA in non-intact families. Unexpectedly, among left-behind students with single-parent migration in intact families, the association between anxiety and IA intensified with high social support, despite a lower incidence of IA compared to low-support conditions. Though this finding diverges from prior research positing social support’s related role [85, 86], perceived social support has been demonstrated to positively associate with IA [87]. The aforementioned result could be derived from this population, with one parent out-migrating for work yet in a harmonious parental relationship, encountering childhood adversity with insufficient coping mechanisms. When exposed to prolonged periods of adequate social support, they may exhibit poorer levels of psychological resilience than other adolescents facing an adverse environment for an extended period. The resilience process model [88] suggests this group is more vulnerable to experiencing stress and psychological strain. As anxiety increases, they may turn to excessive Internet use to alleviate negative emotions and manage the reality of difficult circumstances.

### Implications for practice

As the problem of IA develops increasingly severe, as the matter of left-behind secondary school students grows, they need more attention. This study provides several insights into the relationship between the four for preventing and minimizing the risk of IA among the population.

First, in addressing the issue of IA, we propose practical strategies grounded in the three dimensions of social support: subjective support, objective support, and individual utilization. Firstly, subjective support serves to enhance an individual’s sense of self-efficacy. Those with a heightened sense of self-efficacy are better equipped to confront the challenges associated with internet addiction. When individuals perceive themselves as having a defined status and role within society, they are more inclined to participate in life’s activities rather than succumbing to the allure of the internet. As such, it is incumbent upon parents and schools to augment emotional support for this particular demographic, thereby bolstering their subjective perception of receiving external support in their daily academic and personal lives. This strategy aims to prevent internet addiction among left-behind middle school students. Secondly, objective support becomes salient when individuals perceive tangible or actual assistance, including direct material aid or the existence and engagement of social relationships. This form of support can mitigate internet addiction behavior to a significant degree. Schools and communities can contribute by organizing informative lectures, providing left-behind middle school students and their families with crucial knowledge about internet addiction, and promoting healthy recreational activities. Lastly, the concept of individual utilization is critical. It refers to the extent to which individuals avail themselves of social support. A deficiency in social support is often attributable to an individual’s low utilization of available resources. Enhancing this aspect requires concerted efforts from the students themselves, their families, and their schools.

Second, we must underscore the importance of prioritizing the mental health of left-behind middle school students. As these students transition into adolescence, their emotional states often fluctuate, characterized by instability, immaturity, and a tendency towards impulsivity. In the face of life’s conflicts and challenges, they may harbor feelings of dissatisfaction and injustice, leading to impulsive outbursts of anger. Given their nascent judgment and decision-making abilities, they are susceptible to seeking refuge in the digital world of the internet. Hence, it is paramount to provide tailored guidance that is cognizant of their adolescent characteristics, empowering them to bolster their self-control capabilities. Additionally, the organization of extracurricular activities can be a potent tool for nurturing students’ social skills and adaptability. Such activities can foster a sense of camaraderie among peers and stimulate positive peer interactions. This approach provides a safe space for left-behind middle school students to express their emotions freely and vent any negative feelings, ultimately equipping them with the skills to manage and control their emotional states effectively.

Finally, we should give sufficient attention to the influence of family factors on LBC. In the quest to prevent internet addiction, it is incumbent upon parents to adopt a nuanced perspective of the internet rather than resorting to outright rejection. Parents must learn to navigate the online world alongside their children in this digital age. In particular, left-behind middle school students are more prone to internet addiction due to factors such as parental absence, a lack of family interaction, and inadequate parent–child relationships, all of which are a product of their familial environment. Consequently, we propose that by altering family dynamics, enhancing parent–child interactions, and improving parent–child relationships, we can effectively curb internet addiction among left-behind middle school students. This approach will encourage these students to shift their focus toward their real-life experiences and away from the virtual world. Due to the absence of parents caused by parents working outside, the emotional needs of left-behind middle school students for their families deserve more attention. Parents working outside can also communicate more with their children through social software, telephone videos, and other ways to care about their students and lives.

### Strengths and limitations

The study is not devoid of limitations. The first limitation of the study lies in the following ways: the study design and selection of study variables. The study design: 1) the research originally relies on a cross-sectional survey to appraise the correlation between IA and its determinants among left-behind secondary students, which does not establish cause and effect. Future studies could adopt a longitudinal approach to clarify the sequence of these relationships. 2) The reliance on self-reported data, despite being from a large sample size and pre-tested in Chongqing City, could introduce bias. Future research might incorporate interviews and alternative survey methods to enhance the reliability of findings. The selection of research variables: The study assessed IA using the IAT. While IAT stands as one of the most frequently utilized tools, boasting high levels of efficacy and reliability, it also harbors limitations. The IAT offers a comprehensive assessment of internet usage patterns, including online gaming, e-commerce, and social media engagement. However, our broad scale may fail to capture the unique associations these activities have for each individual. For future investigations, the integration of more specialized measures of internet addiction, particularly those that resonate with the high school population, such as Internet gaming disorder and problematic smartphone use, would be advantageous. This methodological refinement would indisputably enrich the research by adding layers of complexity and subtlety. The second limitation concerns that the research focuses on a culturally specific cohort, potentially influencing the reported levels of IA in the following ways: the study only comprises students from a specific region, therefore, its findings may not be universally applicable. Expanding future research to include a diverse array of participants from various countries or cultural backgrounds could offer broader insights.

Despite its limitations, this research presents significant strengths. It comprises a broad cross-sectional analysis of 5,290 secondary school students in the southeast region of Chongqing, including 2,553 left-behind students. To our knowledge, it stands out as one of the few studies into how anxiety, social support, and family types association with IA among left-behind secondary students, utilizing a vast dataset. Moreover, the study lays out practical recommendations, leveraging the relationship among these elements to propose a variety of targeted countermeasures for systematic intervention or formal diagnosis and treatment of at-risk students.

## Conclusions

The findings of this research emphasize that the anxiety significantly contributes to IA among left-behind secondary students. In addition, it indicates the significant moderating effect of social support in this dynamic, alongside the effect of family types. There appears a pressing need for effective, tailored intervention strategies for IA that consider the social support and family types specific to left-behind secondary students. Despite its limitations, this analysis sheds light on strategies for preventing IA in this population, emphasizing the critical role of social support and the functioning of families.

## Data Availability

The datasets generated and/or analysed during the current study are not publicly available due to privacy or ethical restrictions but are available from the corresponding author on reasonable request.
